# The *in vitro* Effect of Fibers With Different Degrees of Polymerization on Human Gut Bacteria

**DOI:** 10.3389/fmicb.2020.00819

**Published:** 2020-05-15

**Authors:** Miao Chen, Bei Fan, Shujun Liu, Khandaker Md Sharif Uddin Imam, Yingying Xie, Boting Wen, Fengjiao Xin

**Affiliations:** ^1^Laboratory of Biomanufacturing and Food Engineering, Institute of Food Science and Technology, Chinese Academy of Agricultural Sciences, Beijing, China; ^2^Institute of Food Science and Technology, Chinese Academy of Agricultural Sciences, Beijing, China; ^3^Key Laboratory of Agro-products Quality and Safety Control in Storage and Transport Process, Ministry of Agriculture and Rural Affairs, Beijing, China

**Keywords:** *in vitro* fermentation, polymerization degree, dietary fiber, human gut bacteria, short-chain fatty acids, microbial communities

## Abstract

Human gut bacteria contribute significantly to human health and several studies have evaluated the effects of dietary fibers on human gut bacterial ecology. However, the relationship between different degrees of fiber polymerization and human gut bacteria is unknown. Here, we analyzed three fiber substrates with different degrees of polymerization, namely carboxymethylcellulose, β-glucans, and galactooligosaccharides. To probe the *in vitro* influence of the degree of polymerization of the fiber on human gut bacteria, we measured the pH, air pressure, and short-chain fatty acid content of fecal fermentation supplemented with these fiber substrates, and sequenced the 16S ribosomal RNA genes of the microbial community in the fiber-treated fermentations. The butyric acid concentration was shown to decline with decreasing degree of polymerization of the fiber. Illumina Miseq sequencing indicated that the degree of polymerization might have an influence on human gut microbial diversity and abundance. Principal coordinate analysis unveiled a relationship between the degree of fiber polymerization and the gut bacterial community. Specific microbiota operational taxonomic units (OTUs) within the genera *Escherichia-Shigella, Fusobacterium*, and *Dorea* were proportional to the degree of fiber significantly, whereas OTUs within the genera *Bifidobacterium*, *Streptococcus*, and *Lactobacillus* were inversely correlated with the degree of polymerization. Correlation analysis between the fiber degree of polymerization and gut bacteria may demonstrate the effect of fibers on gut microbiota, and subsequently, on human health.

## Introduction

The human gut bacterial population is host-specific, constantly changing throughout an individual’s lifetime and prone to influence or harm by both exogenous and endogenous modification (Sekirov et al., 2010). Therefore, human gut bacteria evolve over time with changes in diet and overall health (Hooda et al., 2012). Recent food science research has demonstrated that dietary fiber has an influence on human gut bacterial ecology. The microbial ecosystem can be disrupted by low-fiber diets, causing chronic inflammation, which leads to the eradication of essential microbial taxa. The average total dietary fiber intake in Chinese adults is far below the recommended daily intake, which can lead to chronic metabolic conditions (Wang et al., 2014), such as type 2 diabetes, cardiovascular disease, and obesity (Kaoutari et al., 2013; Gentile and Weir, 2018; Sanna et al., 2019).

The degree of polymerization (DP) impacts the physicochemical properties and physiological effects of fiber type. Existing research indicates that non-digestible oligosaccharides promote health and treat diseases (Swennen et al., 2006). Sulfated galactooligosaccharides with a low DP (even pentasaccharides) affect fibroblast growth factor FGF-2, potently inhibiting angiogenesis (Bruno-Barcena and Azcarate-Peril, 2015). Inulin-type fructans with a higher DP exert a greater probiotic effect *in vitro* than inulin-type fructans with a lower DP (Van De Wiele et al., 2007). However, the influence of DP on human gut bacteria warrants assessment (Verspreet et al., 2016).

To evaluate the effects of dietary fibers of different DPs on intestinal bacteria, three substrates common in daily life with different DP values, namely carboxymethylcellulose (CMC), β-glucans (BG), and galactooligosaccharides (GOS), were used to assess the influence of fiber DP on gut bacteria. CMC has a DP of 100∼2000, is deemed “generally regarded as safe (GRAS),” and is used in various foods at up to 2.0%. Regular consumption of the dietary emulsifier CMC impacts mouse gut microbiota, facilitating colitis and metabolic syndrome (Chassaing et al., 2015), and is hypothesized to contribute to the increased incidence of chronic gut inflammatory diseases (Viennois et al., 2017). However, the effect of CMC on human gut bacteria has not been studied (Swidsinski et al., 2009). BG, whose DP is 17∼22, is beneficial to intestinal bacteria and is used as a complementary and immunomodulatory agent or adjuvant therapy for breast and liver cancers (Sveinbjo Rnsson et al., 1998). However, how BG regulates gut bacteria is not clear (Jayachandran et al., 2018). GOS, whose average DP is ∼3, promotes both pro- and anti-inflammatory cytokine capacities, which may help suppress *Salmonella typhimurium* colonization (Searle et al., 2012). GOS induces the yield of sundry short-chain fatty acids (SCFAs) and increases several species of *Bifidobacterium* (Ladirat et al., 2014). However, GOS digestion by intestinal bacteria has not been well characterized.

Utilizing *in vitro* models to recognize the influence of different DPs on human gut microbiota can provide powerful information (Fehlbaum et al., 2018). *In vitro* fermentation on human intervention studies have yielded results corresponding to *in vitro* results (Sasaki et al., 2018). In addition, *in vitro* experiments do not have the same ethical constraints as *in vivo* human trials, allowing dynamic sampling to better comprehend microbial activity.

Therefore, this study investigated the influence of fiber substrates with differing DPs on human gut bacteria by performing *in vitro* batch fermentations with CMC, BG, and GOS as substrates. Physicochemical properties including gas production, pH, and SCFA yield (Venema and Abbeele, 2013) were measured. Changes in gut microbiota community structure and composition in the CMC-, BG-, and GOS-treated fermentations were analyzed by Illumina MiSeq high-throughput sequencing using the V3-V4 region of the 16S rRNA gene. The obtained results may provide insight into the relationship between fiber DP and gut bacteria to help develop strategies to determine safe and effective doses in foods.

## Materials and Methods

### Substrates and Fecal Inoculum Collection and Preparation

The three substrates used were CMC (NOVON, Beijing, China), purity ≥ 99%; BG (YIKANG, Zhangjiakou, Hebei, China), purity ≥ 80%; and GOS (Solarbio, Beijing, China), purity ≥ 57%. Substrates with different DPs have different molecular weights. Therefore, we examined the molecular weights of CMC and GOS, determined their average DP and studied the effects of DP difference on the *in vitro* fermentation of intestinal bacteria. The molecular weight of CMC and GOS was determined by DAWN HELEOSII laser scattering (Wyatt Technology Corporation, Santa Barbara, CA, United States), using a Shodex OHPak SB806M HQ column (SHOWA DENKO K.K., Tokyo, Japan). The CMC polymerization degree was calculated to be 2194, the BG polymerization degree was calculated to be 17–22, and the GOS polymerization degree was calculated to be 1.29.

This study was carried out in accordance with the recommendations of provisions on Article 11 of the “Ethics Review Methods for Human-Related Biomedical Research (Draft for Soliciting Opinions)” (National Health and Family Planning Commission of China). The protocol was approved by the Human Research Ethics Committee of Institute of Food Science and Technology, Chinese Academy of Agricultural Sciences. All subjects gave written informed consent in accordance with the Declaration of Helsinki.

Human fecal samples were obtained from 10 healthy donors (5 females and 5 males) who were not treated with antibiotics for more than 2 months before the trial. All participants were recruited by the following inclusion criteria: aged between 22 and 36 years old, Chinese citizens, non-smoking, and healthy. Exclusion criteria included clinically significant deviations from normal depending on investigator judgment; history or suspicion of diabetes, liver disease, kidney disease, or having a food allergy; or taking dietary fiber supplements or lipid-lowering drugs. The inoculum was prepared by suspending the fecal sample in 0.1 M phosphate-buffered solution (pH 6.5, 0.2 M NaH_2_PO_4_ and 0.1 M Na_2_HPO_4_) supplemented with 1.0% L-ascorbic acid. The concentration of the fecal suspension was 10% (wt/vol).

### *In vitro* Fermentation

*In vitro* fermentation was performed in a penicillin vial fermenter with ten parallel and independent vials. Each vial contained 5 mL of YCFA medium (Schwab et al., 2017) and 1% substrate (wt/vol). A control (CK) was prepared with no substrate. A mixture of N_2_ and CO_2_ gases (80:20; 0.02∼0.04 MPa) was used to induce anaerobic conditions, and the mixture was sterilized using a 0.2-μm PTFE membrane. Each vial was autoclaved at 121°C for 20 min. Using a 1 mL injection syringe, 0.5 mL fecal suspension was inoculated into the corresponding vial [1% (wt/vol)] and anaerobic fermentation cultivation was conducted at 37°C. After 24 h of cultivation, air pressure was measured by a BMP-Test System pressure gauge (WAL Mess- und Regelsysteme GmbH, Oldenburg, Germany) and the pH value of the supernatant was measured using a compact pH meter (Model B-212, Horiba, Japan). Samples were harvested for the detection of SCFA production and bioinformatics analysis.

### SCFA Determination

Short-chain fatty acids (acetic acid, propionic acid, and butyric acid) and branched SCFAs (isobutyric acid, valeric acid, and isovaleric acid) were analyzed by gas chromatography. Harvested samples (500 μL) were mixed with 100 μL crotonic acid, incubated at −20°C for 12 h, centrifuged (16060 × *g* for 3 min), and 100 μL was injected into GC-9720 (Zhejiang Fuli Analytical Instrument Co., Ltd) with an HP-FFAP column (30 m × 0.25 mm × 0.25 μm; Agilent Technologies Inc., Santa Clara, CA, United States). The following gas chromatography conditions were used: feed inlet parameters: 250°C, sample injection: 1 μL; carrier gas type: N_2_, blow sweep flow rate 3.0 mL/L, shunt ratio 5:1; column flow rate: 2.3 mL/min; column box parameters: starting temperature: 75°C, warming rate: 20°C/min rose to 180°C, maintain 1 min, 40°C/min rose to 220°C, maintain 1 min, complete procedure time 8.05 min; FID detector parameters: temperature 250°C, tail blowing type N_2_; tail blowing gas flow: 30 mL/min; hydrogen flow: 40 mL/min, air flow: 400 mL/min. Using external calibration curves, acetic acid, propionic acid, butyric acid, isobutyric acid, valeric acid, and isovaleric acid were quantified in the samples.

### Microbial Sequencing Data Acquisition

For analyzing the influence of different DPs on the microbiota communities in the *in vitro* fermentation, genomic DNA from 40 samples was extracted using the E.Z.N.A.^®^ Soil DNA Kit (Omega Bio-Tek, Norcross, GA, United States) following the manufacturer’s protocols. The V3-V4 hypervariable regions of the bacterial 16S rRNA genes were amplified with bacteria-universal primers 338F (5′-ACTCCTACGGGAGGCAGCAG-3′) and 806R (5′-GGACTACHVGGGTWTCTAAT-3′) using a thermocycler PCR system (GeneAmp 9700, ABI, United States). The pooled purified amplicons were sent to Shanghai Majorbio Bio-Pharm Technology Co., Ltd. (Shanghai, China), and sequenced on an Illumina Miseq sequencing platform (Illumina, San Diego, CA, United States). All of the sequences were clustered into Operational Taxonomic Units (OTUs) based on a 97% identity threshold by the SILVA database (Quast et al., 2013). The raw reads used in this study have been deposited into the NCBI Sequence Read Archive (SRA) (Accession number: PRJNA573754).

### Statistical Analysis

Data processing and statistical analyses were performed using GraphPad Prism version 7.0.4 for Windows (GraphPad Software, La Jolla, CA, United States^1^). Principal coordinates analysis (PCA) based on R language was used to study the similarity or difference in the community composition of the three treatments, including β-diversity analysis. Independent-samples Kruskal-Wallis test was performed to evaluate the significant difference (*P* < 0.05 or *P* < 0.01) in species among the three substrates. Canonical correspondence analysis (CCA) was performed to determine the correlation between community distance matrices and environmental factors (acetic acid, propionic acid, and butyric acid). Correlation Heatmap Analysis was performed using the spearman rank correlation coefficient, visually representing the data in a defined shade of color.

## Results

### Effect of DP on Air Pressure and pH

**FIGURE 1:**
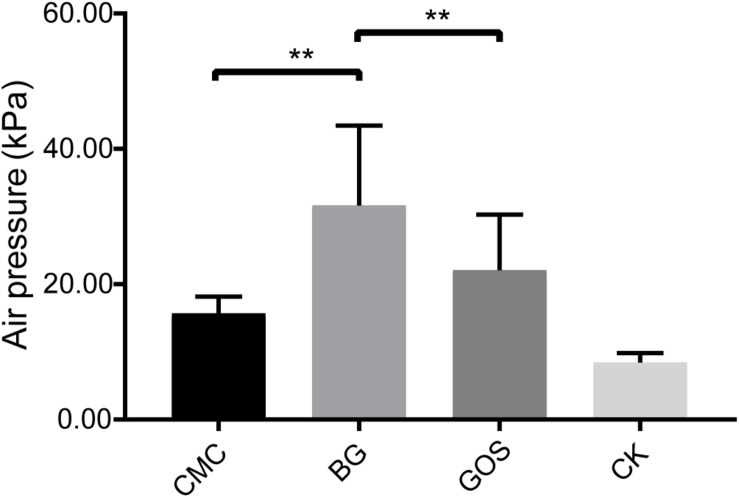
Air pressure of each treatment fermentation. The significant difference in air pressure among the carboxymethylcellulose (CMC), β-glucans (BG), galactooligosaccharides (GOS), and the control (CK) treatments was calculated using the paired *t*-test with **P < 0.01 compared to the control.

The air pressure of the fermentation with CMC, GOS, and BG as substrates was compared with that of CK using a paired *t*-test. Results in Figure 1 show that the air pressure of the fermentations with the three substrates increased. Significantly higher air pressure was obtained in the BG-treated fermentation than in the CMC- and GOS- treated fermentations (*P* = 0.0012 and 0.0027, respectively). The air pressure of the BG-treated fermentation was 31.65 MPa, which was nearly twice that of the CMC-treated fermentation (15.68 MPa).

**FIGURE 2:**
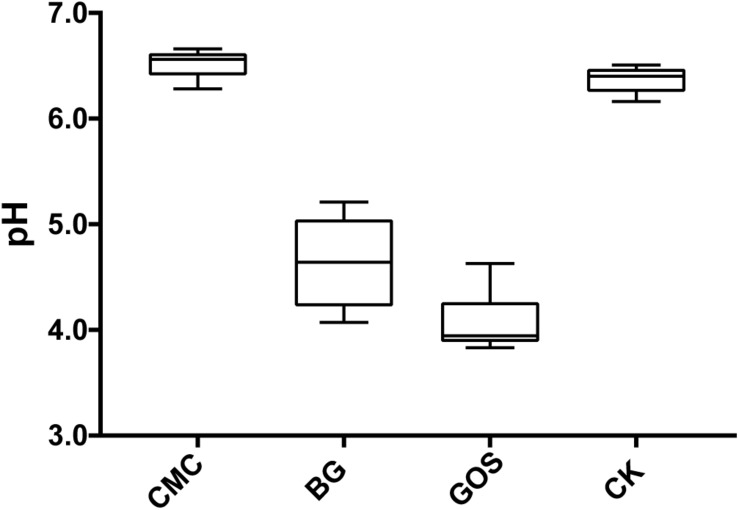
pH value of each treatment fermentation. The pH after fermentation with carboxymethylcellulose (CMC), β-glucans (BG), galactooligosaccharides (GOS), and control (CK).

Results in Figure 2 show that the pH value decreased with decreasing fiber DP during fermentation. The pH value of the CMC-treated fermentation was close to that of CK, which was 6.5. However, the pH values of the BG- and GOS-treated fermentations were 4.6 and 4.1, respectively.

### Effect of DP on SCFAs

**FIGURE 3:**
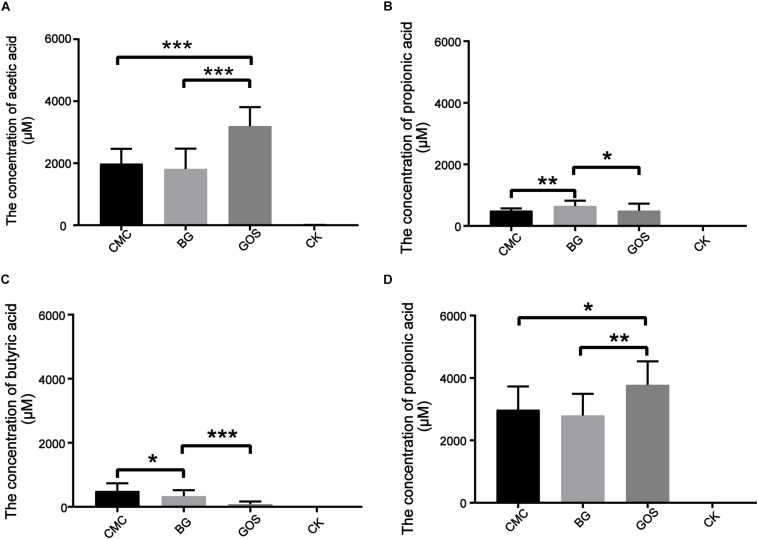
Production of SCFAs after 24 h of *in vitro* fermentation. The figures represent concentration (μM) of acetic acid **(A)**, propionic acid **(B)**, butyric acid **(C)**, and total SCFAs **(D)** in each treatment. Significant differences in the production of SCFAs in each treatment fermentation and the control were calculated using the paired *t*-test with *P < 0.05, **P < 0.01, and ***P < 0.001 compared to the control. Carboxymethylcellulose (CMC), β-glucans (BG), galactooligosaccharides (GOS), control (CK), short-chain fatty acids (SCFAs).

Short-chain fatty acids are universally considered to be beneficial for human health. To study the effects of *in vitro* fermentation on the production of SCFAs in three different DP treatments, the concentrations of acetic acid, propionic acid, butyric acid, isobutyric acid, valeric acid, and isovaleric acid were measured. The data were compared with those of CK using the paired *t*-test. SCFA generation after 24 h of *in vitro* fermentation is shown in Figure 3. Production of acetic acid, propionic acid, and butyric acid increased after the addition of the three substrates. As shown in Figure 3A, the acetic acid concentration from the GOS-treated fermentation (3197 μM) was nearly 60% more than that of the CMC- and BG-treated fermentations (1988 and 1818 μM, respectively). Similar to the results for air pressure, the propionic acid concentrations in the BG-treated fermentation were significantly different from those in the CMC- and GOS-treated fermentations (*p* = 0.0052 and 0.014, respectively) (Figure 3B). The butyric acid concentrations varied among CMC-, BG-, and GOS-treated fermentations (Figure 3C) with a trend of CMC (485 μM) > BG (450 μM) > GOS (292 μM). Total acid concentrations of the six acid types in the CMC-, BG-, and GOS-treated fermentations were 2980, 2795, and 3779 μM, respectively (Figure 3D). The production of isobutyric, valeric, and isovaleric acids was less than 150 μM. All of the metabolomics measurements for each sample are provided in Supplementary Table S1.

### Effect of DP on Microbiota Communities

**FIGURE 4:**
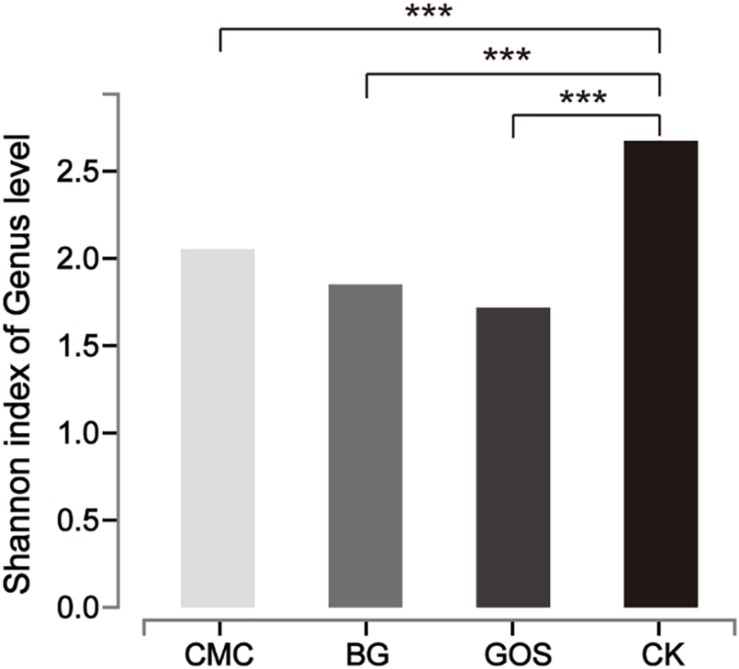
Shannon diversity index of microbial communities from each substrate fermentation. Significant differences were determined between each substrate [Carboxymethylcellulose (CMC), β-glucans (BG), galactooligosaccharides (GOS)] fermentation and the control (CK) using the Student’s *t*-test with ***P < 0.001.

Each of the three fiber types (CMC, BG, and GOS) and CK were fermented using 10% fecal dilution from ten healthy donors. The microbiota communities were analyzed with 16S rRNA gene amplicons. A total of 3,596,733 sequences were generated from 40 samples, and after quality and chimera checking and removal of low-quality reads, a total of 1,800,373 valid sequences were obtained (Supplementary Table S2). All of the cleaned sequences were clustered into operational taxonomic units (OTUs) that shared ≥ 97% sequence identity (Edgar, 2013). The minimum read number was adopted to subsample sequences from all other samples for comparing the treatments and control samples at the same sequencing level (Supplementary Table S3). After subsampling to equal sequencing depth, 27,749 reads per sample were used for further analyses. The rarefaction curve (Supplementary Figure S1) tended to be flat and the amount of sequencing data was large enough to reflect most of the microbial diversity information in samples. The Shannon diversity index of all intestinal bacteria was calculated (Figure 4). By comparing the diversity of each substrate with that of the control, we obtained significant differences in the estimators of community Shannon diversity between each substrate (2.05, *P* = 0.0003 for CMC-treated fermentation; 1.85, *P* = 0.0002 for BG-treated fermentation; 1.72, *P* = 0.00003 for GOS-treated fermentation respectively) and CK (2.67), indicating that a lower microbiota diversity was induced with the addition of the three substrates.

**FIGURE 5:**
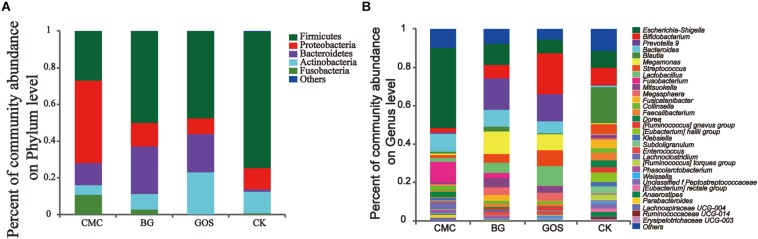
Community abundance after treatment with the three substrates and the control. The relative abundance at the phylum level **(A)** and the genus level **(B)** in the three treatments [carboxymethylcellulose (CMC), β-glucans (BG), galactooligosaccharides (GOS)] and the control (CK).

To evaluate the relationship between the potential benefit of gut bacteria and DP, the bacterial communities from the CMC-, BG-, and GOS-treated and CK *in vitro* fermentations were analyzed at the phylum and genus levels. At the phylum level (Figure 5A), the trend of *Actinobacteria* abundance was proportional to fiber DP, whereas that of *Proteobacteria* was inversely proportional to fiber DP. The dominant bacterial communities in CK were *Firmicutes*, *Proteobacteria*, *Bacteroides*, and *Actinobacteria*, whose relative abundance accounted for 97.4% of the total bacterial communities in the BG- and GOS-treated fermentations, and 89.2% in the CMC-treated fermentation because of the higher relative abundance of *Fusobacteria*. The relative abundance of *Bacteroides* dramatically increased with the addition of the three substrates. *Proteobacteria* abundance increased with CMC treatment. A decrease in the relative abundance of *Firmicutes* was observed in the three treatments compared to that in CK.

At the genus level (Figure 5B), the relative abundance of *Escherichia-Shigella*, *Fusobacterium*, *Dorea*, and *Klebsiella* was proportional to fiber DP, whereas the relative abundance of *Bifidobacterium*, *Streptococcus*, and *Lactobacillus* was inversely proportional to fiber DP. The alpha-diversity values of microbiota in CK were more abundant than those in the three fiber treatments. The dominant bacterial community in CK was *Blautia*, which decreased after treatment with the three fibers. The relative abundance of *Bacteroides* greatly increased after treatment with the three fibers. CMC treatment increased the relative abundance of *Escherichia-Shigella* compared to that in CK. *Fusobacterium* accounted for approximately 10% of the community abundance after CMC-treated fermentation but accounted for only 2.7 and 0% after BG and GOS treatments, respectively. Additionally, with the addition of BG and GOS, the relative abundance of *Prevotella_9* and *Megamonas* was enhanced compared to that under the CMC treatment and CK. The relative abundance of *Bifidobacterium* in the GOS-treated fermentation was obviously greater than that in the BG- and CMC-treated fermentations.

**FIGURE 6:**
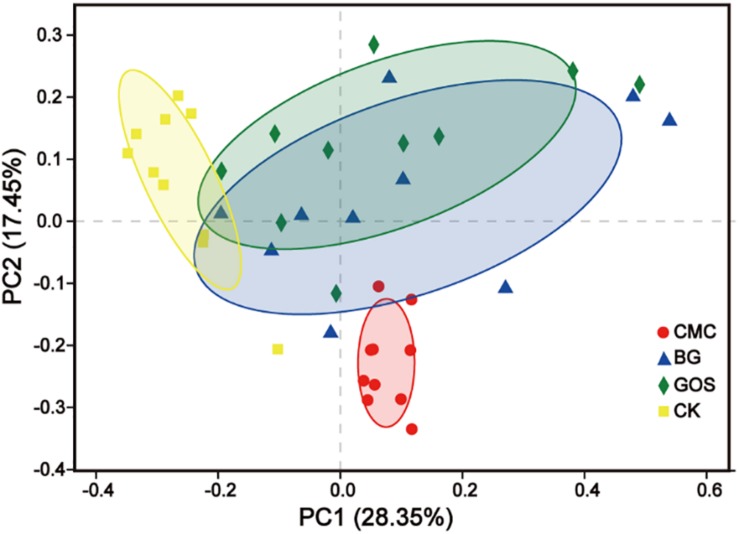
Principal Coordinate Analysis (PCoA) based on Weighted UniFrac dissimilarity between fermentation of carboxymethylcellulose (CMC), β-glucans (BG), galactooligosaccharides (GOS), and the control (CK).

Through Principal Coordinate Analysis (PCoA) using the weighted UniFrac distance matrix at the OTU level, spatial separation and clustering of the fermentation bacteria from the CMC- and CK-treated fermentations were clear. The microbial community was similar in BG- and GOS-treated fermentations as part of their microbial bacteria overlapped. As shown in Figure 6, a unique microbial bacterial distribution from top to bottom with increasing values of fiber DP was observed, which suggests that fiber DP affects the gut microbiota.

**FIGURE 7:**
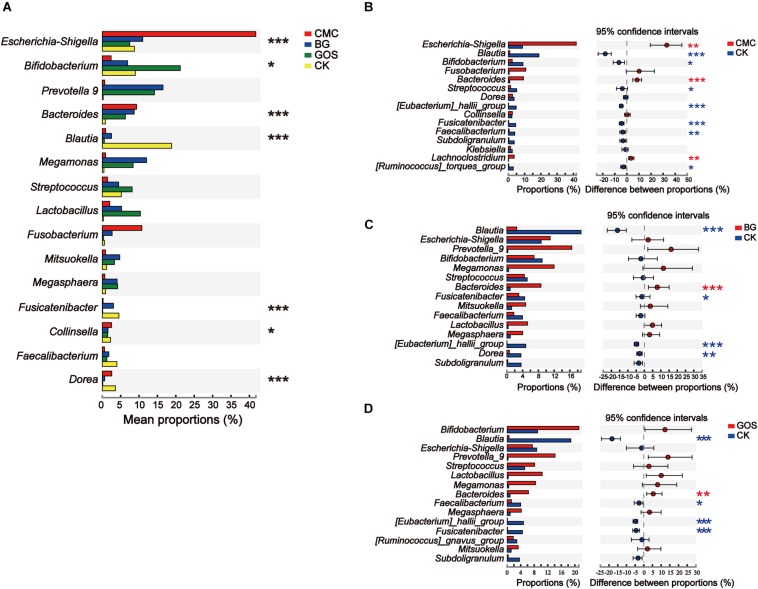
Significant differences between the three substrates and the control. Analysis of phylotypes significantly different among **(A)** the three substrates and the control and **(B–D)** between each fermentation and the control at the genus level. The significant difference in microbiota among carboxymethylcellulose (CMC), β-glucans (BG), and galactooligosaccharides (GOS) fermentation and the control (CK) was calculated using the Kruskal–Wallis test (*P < 0.05, **P < 0.01, and ***P < 0.001) and the significant difference in microbiota between carboxymethylcellulose (CMC) fermentation and the control (CK) **(B)**, β-glucans (BG) fermentation and the control (CK) and **(C)**, galactooligosaccharides (GOS) fermentation and the control (CK) **(D)** were calculated using the Wilcoxon test with *P < 0.05, **P < 0.01, and ***P < 0.001.

The genera with significant differences by the Kruskal–Wallis *H* test in each treatment are shown in Figure 7. The bacteria with high relative abundance bacteria were *Escherichia-Shigella* (*P* = 0.0007), *Bifidobacterium* (*P* = 0.0322), *Bacteroides* (*P* = 0.0002), *Blautia* (*P* = 0.00003), *Fusicatenibacter* (*P* = 0.0001), *Collinsella* (*P* = 0.0388), and *Dorea* (*P* = 0.00003), which varied significantly between the three fiber treatments and CK (Figure 7A). As shown in Figures 7B–D, CMC, BG, and GOS treatment can stimulate the growth of *Bacteroides*, whereas growth of *Blautia* (*P* = 0.0002 for CMC-treated fermentation; *P* = 0.0002 for BG-treated fermentation; *P* = 0.0002 for GOS-treated fermentation, respectively), *Eubacterium_hallii_group* (*P* = 0.0002 for CMC-treated fermentation; *P* = 0.0002 for BG-treated fermentation; *P* = 0.0002 for GOS-treated fermentation, respectively), and *Fusicatenibacter* (*P* = 0.0002 for CMC-treated fermentation; *P* = 0.0376 for BG-treated fermentation; *P* = 0.0002 for GOS-treated fermentation, respectively) was significantly impaired. There were also unique microbiota changes in each treatment. After CMC treatment, the abundance of *Escherichia-Shigella* (*P* = 0.0010) and *Lachnoclostridium* (*P* = 0.0017) was significantly increased, whereas *Bifidobacterium* (*P* = 0.0233), *Streptococcus* (*P* = 0.0258), *Fecalibacterium* (*P* = 0.0073), and *Ruminococcus_torques_group* (*P* = 0.0173) were significantly decreased (Figure 7B). In the BG treatment group, *Dorea* significantly decreased (*P* = 0.0041) (Figure 7C), while in the GOS treatment group, *Fecalibacterium* significantly decreased (*P* = 0.0312) (Figure 7D). Some microbiota showed a higher relative abundance, such as *Fusobacterium* in CMC, *Prevotella*_9 in BG, and *Lactobacillus* and *Megamonas* in GOS treatment, which differed distinctly from those in CK. However, this difference was not significant because the individual variations in the microbial communities from each fiber treatment were greater than those between groups (Supplementary Figure S2).

### Environmental Factor Correlation Analysis

**FIGURE 8:**
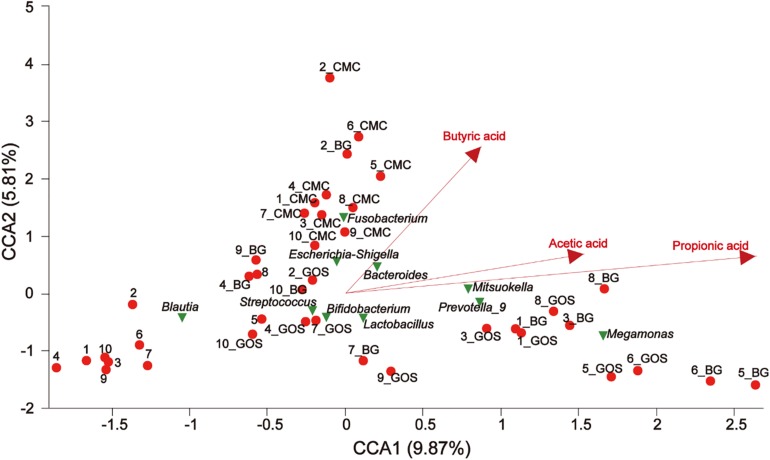
The relationship between environmental factors, samples, and microflora. The dots of different colors or shapes in the figure represent sample treatments and genus level microbiota (top 10); the red arrow represents the quantitative environmental factor, and the length of the environmental factor arrow can represent the degree of influence of the environmental factor on the species data (interpretation). The size of the environmental factor arrow represents positive and negative correlation (sharp angle: positive correlation; obtuse angle: negative correlation; right angle: no correlation); projection from the sample point to the arrow of the quantitative environmental factor is the projection point distance. The distance of the origin represents the relative influence of environmental factors on the distribution of sample communities.

Canonical correspondence analysis (CCA) revealed a connection between the community composition of microbiota at the genus level and different environmental factors for treatments with fibers of different DPs. CMC, BG, and GOS greatly affected the bacterial community composition. The results of CCA analysis (Figure 8) showed that CMC and genus *Escherichia-Shigella* were closely associated with the butyric acid yield. BG and GOS treatments remarkably affected the production of propionic acid and had an osculatory correlation with *Megamonas*, *Prevotella_9*, and *Mitsuokella*.

**FIGURE 9:**
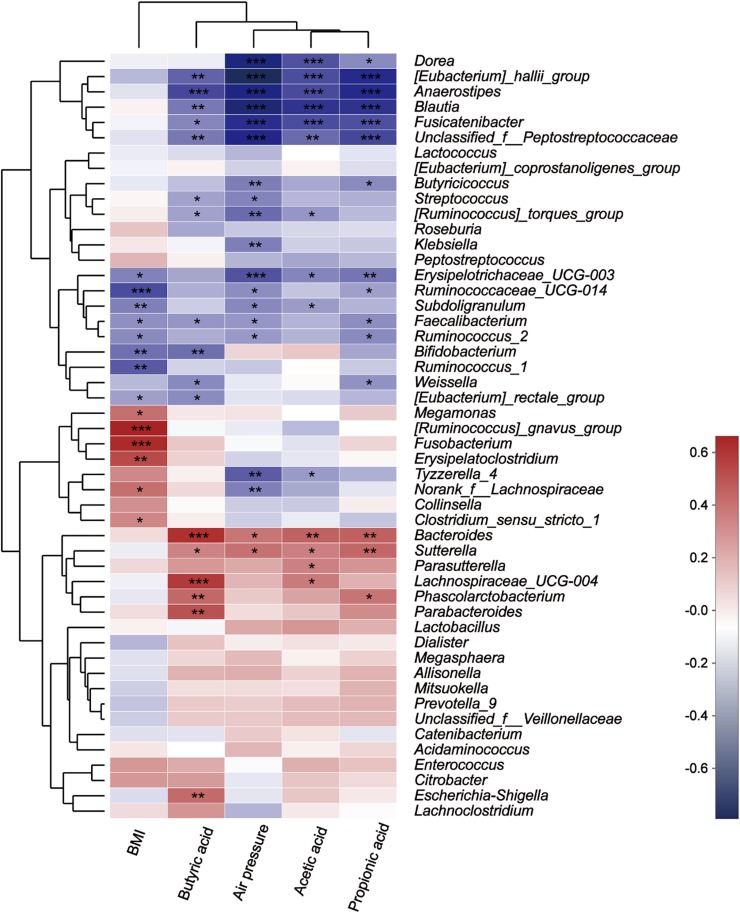
Correlation between microbes at the genus level and environmental variables. The *X*-axis and *Y*-axis are environmental factors and species, respectively, and the *R*-value and the *P*-value are obtained by calculation. The *R*-value is shown in different colors in the figure. If the *P*-value is less than 0.05, it is marked with a *, and the right legend is a color interval with different *R* values. You can choose to present species and environmental factor clustering trees (such as the left and upper sides). *0.01 < *P* ≤ 0.05, **0.001 < *P* ≤ 0.01, and ***P ≤ 0.001.

The Spearman correlation heatmap was used to analyze the impacts of different environmental factors on the communities of intestinal bacteria after treatment with fibers of different DPs. The results (Figure 9) showed that the intestinal bacterial community structure was simultaneously affected by Body Mass Index (BMI) (Supplementary Table S4), air pressure, acetic acid, propionic acid, and butyric acid. The effect of different microbiota on BMI, air pressure, acetic acid, propionic acid, and butyric acid differed. The relative abundance of [*Ruminococcus*] *gansvus group* (*P* = 0) and *Fusobacterium* (*P* = 0) was significantly positively correlated with BMI. The genera *Ruminococcaceae UCG-014* (*P* = 0), *Bifidobacterium* (*P* = 0.003) and *Ruminococcus 1* (*P* = 0.001) were significantly negatively correlated with BMI. The genera [*Eubacterium*] *hallii group* (*P* = 0, 0, 0.001, and 0), *Anaerostipes* (*P* = 0, 0, 0, and 0), *Blautia* (*P* = 0, 0, 0.005, and 0), *Fusicatenibacter* (*P* = 0, 0, 0.012, and 0), *and Unclassified f Peptostreptococcaceae* (*P* = 0.002, 0, 0.005, and 0) were negatively correlated with SCFA (acetic acid, propionic acid, and butyric acid) concentration and air pressure in turn. Additionally, the butyric acid concentration was significantly negatively correlated with *Bacteroides* (*P* = 0), *Lachnospiraceae UCG-004* (*P* = 0), *Parabacteroides* (*P* = 0.001), *Phascolarctobacterium* (*P* = 0.007), and *Escherichia Shigella* (*P* = 0.008).

## Discussion

### DP Impacts SCFA Production

Diet has a significant influence on gut bacterial communities and their metabolites, of which the major SCFAs, acetic acid, propionic acid, and butyric acid (De Filippo et al., 2010; Tremaroli and Backhed, 2012; Carmody et al., 2015), contribute greatly to gut health. Prior studies have reported that the host genetics-driven rise in the intestinal yield of butyric acid was related to enhanced insulin response after an oral glucose tolerance test (Sanna et al., 2019). In addition, large DP fractions were shown to be potential substrates to induce restoration of other butyrate-producing bacteria, and butyric acid was only produced after fermentation of the large size-fractions (Ladirat et al., 2014). Combined with the research data, our results indicated that fibers with higher DPs can promote the production of butyric acid. Moreover, the concentrations of propionic acid and acetic acid were highest with BG- and GOS-treated fermentations, respectively, which concurred with results from previous studies (Davis et al., 2011; Carlson et al., 2017).

### DP Impacts Human Intestinal Bacterial Structure and Abundance

Changes in physicochemical properties reflect microbiota activity. Microbiota structure and abundance were altered by the three fiber treatments. In healthy adults, the most prevalent phyla are *Firmicutes*, *Bacteroides*, *Actinobacteria*, and *Proteobacteria*, as was observed in the control (Abdessamad et al., 2013). After treatment with three polysaccharides with different DPs, the trend in abundance variation of *Actinobacteria*, which has been used for antibiotic research for many years (Landwehr et al., 2016), was proportional to DP. The variation trend of *Proteobacteria*, which enhances susceptibility to intestinal damage (Mirpuri et al., 2014), was inversely proportional to DP. The abundance of *Prevotella*, which is particularly common in long-chain carbohydrates or plant-based foods dietary patterns (Wu et al., 2011), in the BG- and GOS-treated fermentations was significantly higher than those in the CMC-treated fermentation and CK. In summary, there is a correlation between fiber DP and probiotic effect.

### DP Induces Significant Differences in Human Gut Bacteria

The abundance of *Escherichia-Shigella* and *Fusobacterium* greatly increased during *in vitro* fermentation using the CMC substrate. Interestingly, *Escherichia-Shigella* induces inflammatory reactions and ulceration as well as bloody or mucoidal diarrhea (Ud-Din and Wahid, 2014). In addition, the relative abundance of another special genus, *Fusobacterium*, was significantly higher during CMC-treated fermentation compared with the other fiber treatments with lower DP. *Fusobacterium* is mainly associated with cancer cells in metastatic lesions (Bullman et al., 2017). Moreover, CMC, which has a higher DP, was used as an inflammation model in previous research (Wen et al., 2014). In summary, a key factor underlying the pro-inflammatory effect of CMC might be the aforementioned bacteria. Previous studies indicated that BG, which has an intermediate DP value among the three substrates, can enhance the abundance of *Bacteroides-Prevotella* and the production of propionic and butyric acids during *in vitro* fermentation (Louis and Flint, 2017), and can decrease the relative abundance of *Dorea*, which is regarded as a pathogenic bacterial genus. Supplementation with GOS, which has the smallest DP value among the three substrates, is considered to exert a “bifidogenic effect” (Davis et al., 2011; Sangwan et al., 2011). Similarly, the relative abundance of *Bifidobacterium* significantly increased in the GOS-treated fermentation. Moreover, a decrease in the abundance of *Fecalibacterium* has anti-inflammatory functions in Crohn disease patients (Sokol et al., 2008).

### Lower DP Produces More Probiotic Effects

Physicochemical properties are closely linked to microbial community structure and abundance. The yield of butyric acid in the CMC-treated fermentation was the highest among the three substrates. Coincidentally, the abundance of *Escherichia-Shigella* was also the highest in the CMC-treated fermentation. Butyric acid is beneficial to enterohemorrhagic *E. coli* by inducing the production of flagella that regulates its motility function (Herold et al., 2009; Tobe et al., 2011). It also promotes virulence factor gene expression in pathogenic *E. coli* and thus, colonization of the colon where levels of butyric acid are the highest (Nakanishi et al., 2009). CMC might induce inflammation as it promotes the production of large amounts of butyric acid by intestinal bacteria, and a large amount of butyric acid can boost the expression of *Escherichia-Shigella* virulence factors, thereby triggering inflammation. Reasonably, *Prevotella spp.*, which are overrepresented in the presence of autoinflammatory diseases (Lukens et al., 2014), showed a low relative abundance in the CMC- treated fermentation.

In BG- and GOS-treated fermentations, the abundance of beneficial genera *Bifidobacterium*, *Prevotella*_9, *Bacteroides*, *Megamonas*, *Lactobacillus*, and *Megasphaera* (Kwon et al., 2018) improved compared to that in CK. Increased abundance of *Bifidobacterium*, an acetic acid producer, is negatively related to diabetes, hepatocellular carcinoma, and non-alcoholic steatohepatitis (Cani et al., 2007; Ladirat et al., 2014). Similarly, in the GOS-treated fermentation, both the acetic acid concentration and abundance of *Bifidobacterium* were the highest among the three fermentation groups (Figures 3A, 5B). *Prevotella*, a commensal bacterium, improves the ability of intestinal bacteria to degrade complex polysaccharides and fibers (Petia et al., 2015). *Bacteroides*, a butyric acid producer (Louis et al., 2004; Shin et al., 2017), *Lactobacillus*, predominantly a lactic acid producer, and *Megasphaera*, a lactic acid and SCFA producer (Luo et al., 2017), induced an acidic environment, thereby decreasing the abundance of *Escherichia-Shigella* (Meng et al., 2017). The pH was close to neutral in the CMC-treated fermentation, which provided *Escherichia-Shigella* with a suitable growth environment.

Overall, BG and GOS have potential benefits for modulating the community structure of beneficial intestinal bacteria. This study provides new insight for studying the effects of dietary fibers with different DPs on intestinal bacteria. The future development of human intestinal bacteria as food additives can also be quickly assessed using *in vitro* fermentation techniques. Specific elements of the enteropathogenic microbiota, such as *Escherichia-Shigella*, dramatically increased during CMC fermentation whereas the majority of the increased microbiota in BG- and GOS-treated fermentations were beneficial to humans. Therefore, the dosage of CMC should be strictly controlled in food. Although interpersonal deviation exists, the data indicate a positive correlation between beneficial bacteria and BG and GOS, indicating that lower fiber DP values induce greater probiotic effects. To follow up, long-term *in vitro* fermentation (Poeker et al., 2018) and metagenome sequencing analysis (Wang et al., 2020) are expected to provide a more in-depth characterization of how fiber DP affects the human gut and host health. *In vivo* experiments for additional information regarding the species of bacteria and the mechanisms underlying their effects are warranted.

## Conclusion

Degree of polymerization is an important indicator of physical and chemical structure of dietary fiber. This study investigate the effect of fibers supplementation with different DP value on *in vitro* growth of undefined bacterial communities representing the human gut microbiota. Changes in the community taxonomic composition were analyzed through 16S metagenomic sequencing, and the metabolic outcomes including the most important production of SCFA were measured after fermentation by gas chromatography.

The pH value, the yield of butyric acid, and the genera *Escherichia-Shigella*, *Fusobacterium*, and *Dorea* were proportional to the DP of fiber significantly, whereas OTUs within the genera *Bifidobacterium*, *Streptococcus*, and *Lactobacillus* were inversely correlated with the DP. The results show that the fibers with high DP value have potential benefits for modulating the microbial community structure to larger relative abundance of butyrate producers. This study is an important contribution to the field of the human gut microbiome.

## Data Availability Statement

The raw reads used in this study have been deposited into the NCBI Sequence Read Archive (SRA) [Accession number: PRJNA573754].

## Ethics Statement

This study was carried out in accordance with the recommendations of provisions on Article 11 of the “Ethics Review Methods for Human-Related Biomedical Research (Draft for Soliciting Opinions)” (National Health and Family Planning Commission of China). The protocol was approved by the Human Research Ethics Committee of Institute of Food Science and Technology, Chinese Academy of Agricultural Sciences. All subjects gave written informed consent in accordance with the Declaration of Helsinki.

## Author Contributions

MC, BF, SL, YX, BW, and FX designed the study. MC and SL conducted *in vitro* fermentation, culturing, measured pH and air pressure, quantified SCFA concentrations and performed DNA extraction. MC conducted physicochemical property analysis, bioinformatics, and all statistical analysis. BW, MC, and FX interpreted the results. MC drafted the manuscript with contributions of BW and FX. BW, KI contributed to Writing–review, and editing. Conceptualization, BW. The visualization, supervision, project administration, and funding acquisition were the responsibility of FX. All authors read and approved the final manuscript.

## Conflict of Interest

The authors declare that the research was conducted in the absence of any commercial or financial relationships that could be construed as a potential conflict of interest.

## Footnotes

1 www.graphpad.com
