# Supplementary material for: The in vitro Effect of Fibers With Different Degrees of Polymerization on Human Gut Bacteria
Source: Front Microbiol. 2020 May 15;11:819. doi: 10.3389/fmicb.2020.00819 (PMC7242623; doi:10.3389/fmicb.2020.00819)
Supplement: Supplementary file 1 [file Data_Sheet_1.doc]

# The *in vitro* Effect of Fibers with Different Degrees of Polymerisation on Human Gut Microbiota

**Supplementary material:**

Table S1. The metabolomics measurements of air pressure, pH, acetic acid, propionic acid, butyric acid, and total acid for each sample

| **Air pressure (MPa)** | | | | |
| --- | --- | --- | --- | --- |
| **Donor NO.** | **CMC** | **BG** | **GOS** | **CK** |
| 1 | 14.30 | 26.60 | 17.00 | 6.70 |
| 2 | 17.90 | 31.70 | 21.40 | 10.00 |
| 3 | 18.60 | 41.50 | 19.20 | 9.80 |
| 4 | 15.50 | 36.00 | 19.00 | 7.70 |
| 5 | 17.00 | 58.90 | 42.00 | 6.30 |
| 6 | 18.70 | 21.00 | 17.90 | 7.30 |
| 7 | 13.00 | 22.90 | 19.70 | 8.40 |
| 8 | 16.90 | 33.30 | 29.10 | 9.70 |
| 9 | 12.90 | 21.90 | 22.90 | 8.80 |
| 10 | 12.00 | 22.70 | 12.40 | 9.80 |
| **pH** | | | | |
| **Donor NO.** | **CMC** | **BG** | **GOS** | **CK** |
| 1 | 6.58 | 4.07 | 3.83 | 6.16 |
| 2 | 6.63 | 5.13 | 4.29 | 6.36 |
| 3 | 6.66 | 4.29 | 3.97 | 6.44 |
| 4 | 6.55 | 4.71 | 3.92 | 6.39 |
| 5 | 6.61 | 4.89 | 4.63 | 6.27 |
| 6 | 6.57 | 4.14 | 4.17 | 6.41 |
| 7 | 6.31 | 5.21 | 3.92 | 6.22 |
| 8 | 6.53 | 4.26 | 4.25 | 6.51 |
| 9 | 6.28 | 4.57 | 3.91 | 6.47 |
| 10 | 6.45 | 5.01 | 3.83 | 6.47 |
| **Acetic acid (μ mol/g)** | | | | |
| **Donor NO.** | **CMC** | **BG** | **GOS** | **CK** |
| 1 | 2271.70 | 1474.00 | 2705.13 | 9.27 |
| 2 | 1311.40 | 2237.90 | 3025.00 | 9.94 |
| 3 | 2540.00 | 2600.50 | 4310.38 | 11.67 |
| 4 | 2343.00 | 2853.10 | 3302.25 | 10.03 |
| 5 | 1924.30 | 963.60 | 3090.38 | 10.41 |
| 6 | 2517.80 | 1085.30 | 3054.13 | 11.68 |
| 7 | 1302.80 | 2157.30 | 4069.50 | 9.26 |
| 8 | 2322.90 | 1319.80 | 3462.38 | 11.85 |
| 9 | 1804.70 | 2068.80 | 2532.75 | 9.78 |
| 10 | 1541.60 | 1421.70 | 2420.50 | 10.82 |
| **Propionic acid (μ mol/g)** | | | | |
| **Donor NO.** | **CMC** | **BG** | **GOS** | **CK** |
| 1 | 509.40 | 669.10 | 371.75 | 1.24 |
| 2 | 423.60 | 547.70 | 405.75 | 0.94 |
| 3 | 573.90 | 811.50 | 713.38 | 1.65 |
| 4 | 569.70 | 536.80 | 351.00 | 1.10 |
| 5 | 489.60 | 830.40 | 673.63 | 1.06 |
| 6 | 616.30 | 978.90 | 744.50 | 1.61 |
| 7 | 450.40 | 543.50 | 279.00 | 0.85 |
| 8 | 511.70 | 632.60 | 882.13 | 2.60 |
| 9 | 393.00 | 407.30 | 283.38 | 2.59 |
| 10 | 431.60 | 511.00 | 291.63 | 3.30 |
| **Butyric acid (μ mol/g)** | | | | |
| **Donor NO.** | **CMC** | **BG** | **GOS** | **CK** |
| 1 | 641.50 | 191.90 | 24.63 | 0.22 |
| 2 | 481.10 | 553.30 | 300.38 | 0.12 |
| 3 | 818.40 | 569.40 | 111.63 | 0.23 |
| 4 | 504.80 | 470.20 | 33.63 | 0.30 |
| 5 | 693.10 | 439.00 | 108.38 | 0.34 |
| 6 | 694.40 | 146.40 | 49.75 | 0.22 |
| 7 | 141.80 | 142.50 | 43.50 | 0.17 |
| 8 | 572.80 | 499.90 | 105.88 | 0.30 |
| 9 | 277.40 | 175.30 | 24.75 | 0.24 |
| 10 | 125.80 | 121.10 | 22.25 | 0.25 |
| **Total acid (μ mol/g)** | | | | |
| **Donor NO.** | **CMC** | **BG** | **GOS** | **CK** |
| 1 | 3422.60 | 2335.00 | 3101.50 | 10.73 |
| 2 | 2216.10 | 3338.90 | 3731.13 | 11.00 |
| 3 | 3932.30 | 3981.40 | 5135.38 | 13.55 |
| 4 | 3417.50 | 3860.10 | 3686.88 | 11.43 |
| 5 | 3107.00 | 2233.00 | 3872.38 | 11.81 |
| 6 | 3828.50 | 2210.60 | 3848.38 | 13.51 |
| 7 | 1895.00 | 2843.30 | 4392.00 | 10.29 |
| 8 | 3407.40 | 2452.30 | 4450.38 | 14.75 |
| 9 | 2475.10 | 2651.40 | 2840.88 | 12.61 |
| 10 | 2099.00 | 2053.80 | 2734.38 | 14.37 |

Table S2. Taxon statistics after quality and chimera checking

| **Amplified Region** | **Samples** | **Sequences** | **Bases(bp)** | **Average length** |
| --- | --- | --- | --- | --- |
| 338F_806R | 40 | 1800373 | 791476775 | 439.62 |

Table S3. Taxonomic profiles for each analyzed sample

Table S4. Donor Body Mass Index

| **Type** | **Range** | **Donor No.** | **BMI** | **BMI average** |
| --- | --- | --- | --- | --- |
| Lean | <18.5 | 1  10 | 18.34  18.29 | 18.32 |
| Normal | 18.5-23.9 | 4  7  8  9 | 21.48  20.43  22.1  23.24 | 21.8125 |
| Fat | 24-27.9 | 2  3  5  6 | 26.73  24.91  24.34  27.78 | 25.94 |

Table S5. Donor Body Mass Index

|  | Acetic acid | Propionic acid | Butyric acid | Air pressure | BMI |
| --- | --- | --- | --- | --- | --- |
| VIF | 2.32 | 2.53 | 1.59 | 2.26 | 1.12 |

Figure S1. Rarefaction curve of Shannon-Wiener of the CMC-, BG-, and GOS-treated fermentations at the genus level.


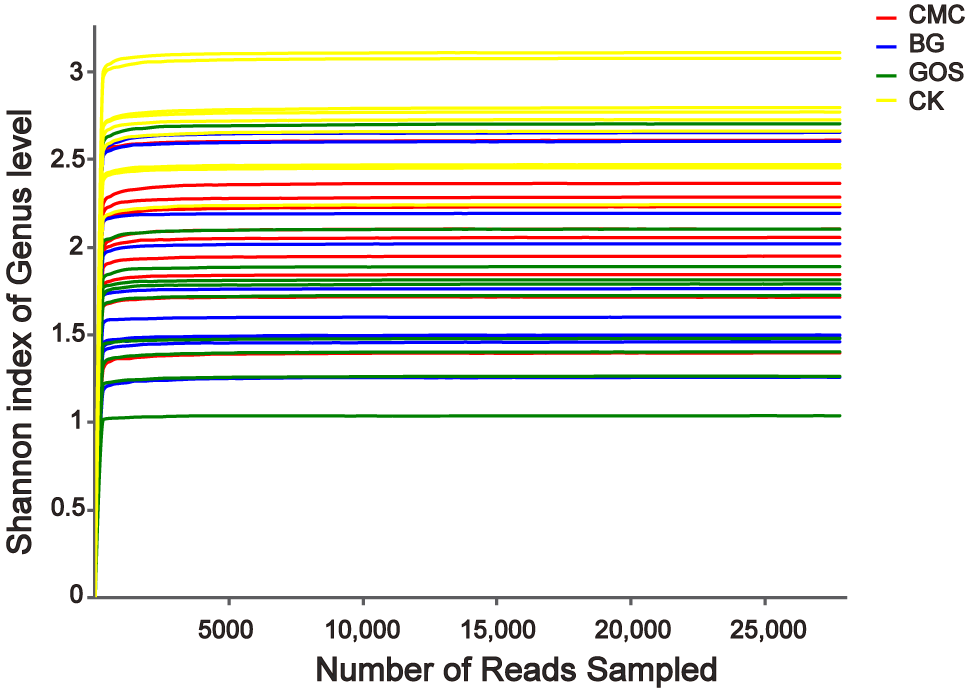


Figure S2. The proportion of sequences of *Fusobacterium* (A), *Prevotella*_9 (B), *Lactobacillus* (C), and *Megamonas* (D) at the genus level of each treatment compared with the control group (CK) in *in vitro* fermentation after 24 h. The gut microbiota originated from ten healthy donors numbered from 1 to 10. The four figures indicate the statistically significant inter-individual difference, while the differences in terms of the proportion of sequences among the four bacterial groups between each treatment (carboxymethylcellulose (CMC), β-glucans (BG), galactooligosaccharides (GOS)) were not statistically significant when compared with the control group (Figure 6B-D).


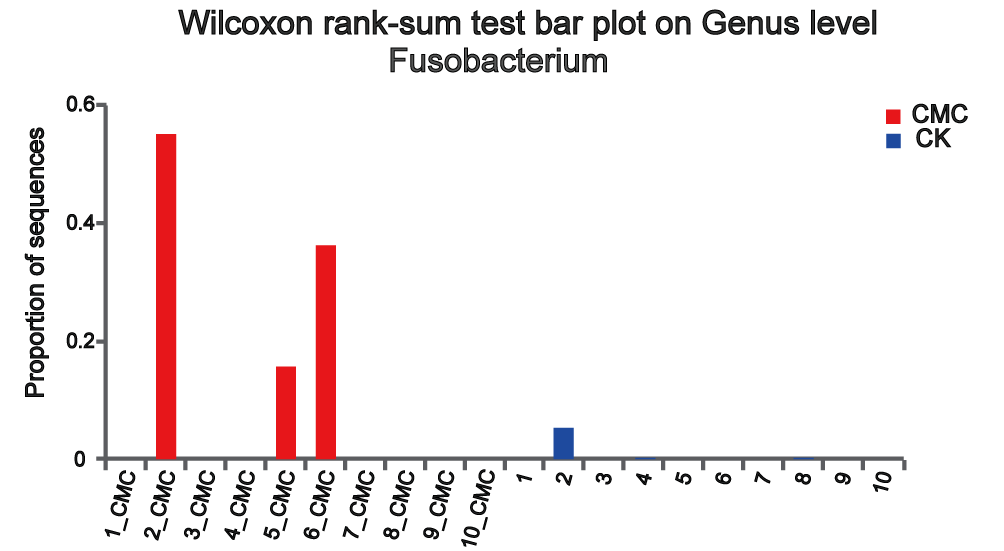


(A)


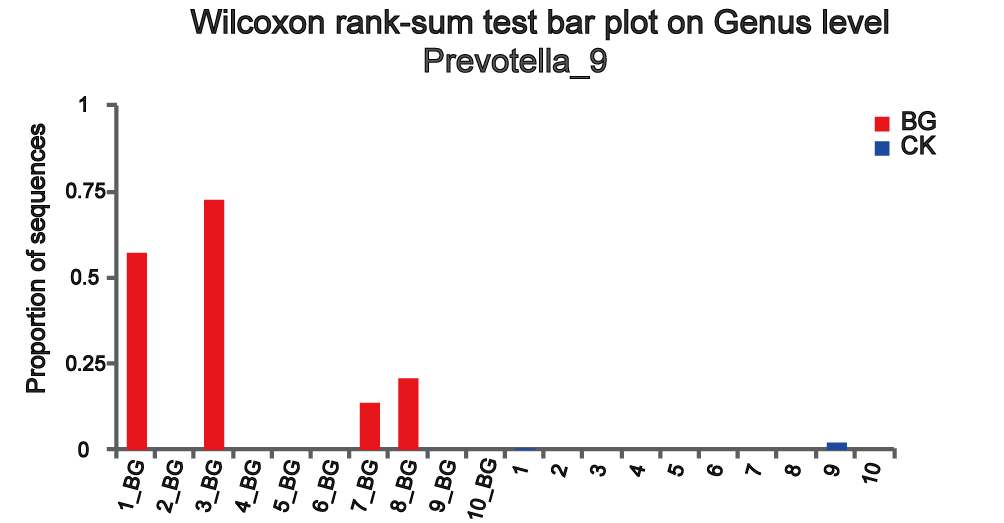


(B)


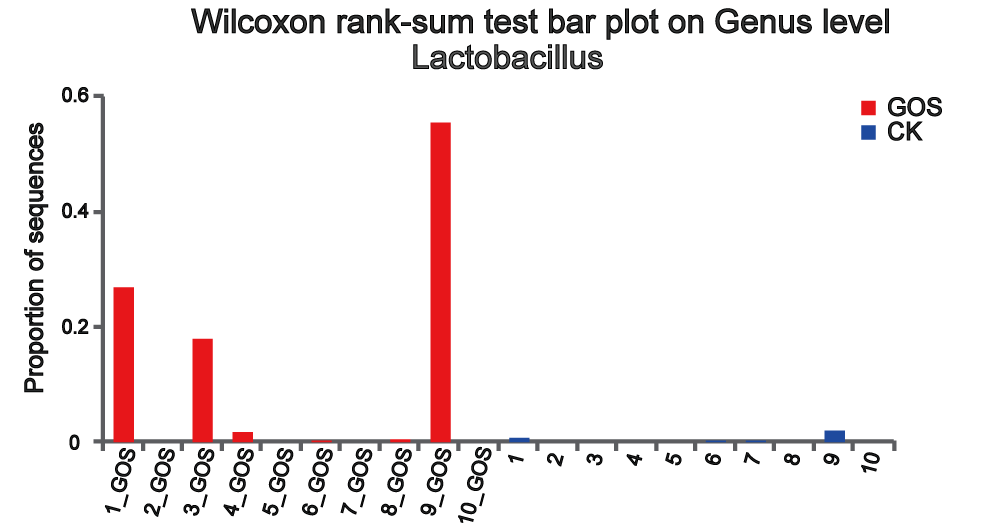


(C)


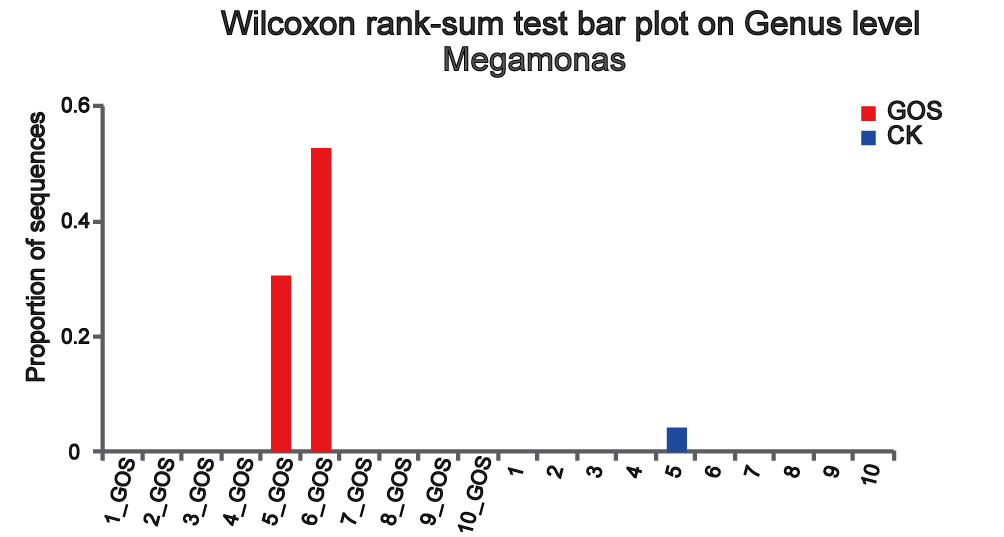


(D)
